# Supplementary material for: Platelet versus fresh frozen plasma transfusion for coagulopathy in cardiac surgery patients
Source: PLoS One. 2024 Jan 17;19(1):e0296726. doi: 10.1371/journal.pone.0296726 (PMC10793891; doi:10.1371/journal.pone.0296726)
Supplement: S1 File — (DOCX) [file pone.0296726.s001.docx]

**Online Supplement**

**Platelet versus fresh frozen plasma transfusion for coagulopathy in cardiac surgery patients**

Jake V Hinton^1^**^*^**, Calvin M Fletcher^2^, Luke A Perry^3,4^, Noah Greifer^5^, Jessica N Hinton^6^, Jenni Williams-Spence^7^, Reny Segal^3,4^, Julian A Smith^8,9^, Christopher M. Reid^7,10^, Laurence Weinberg^1,4^, Rinaldo Bellomo^4,11, 12, 13^

^1^Department of Anaesthesia, Austin Health, Heidelberg, Australia

^2^Department of Anaesthesiology and Perioperative Medicine, The Alfred Hospital, Melbourne, Australia

^3^Department of Anaesthesia and Pain Management, Royal Melbourne Hospital, Parkville, Australia

^4^Department of Critical Care, University of Melbourne, Parkville, Australia

^5^Harvard University Institute for Quantitative Social Science, Cambridge, MA

^6^Department of Orthopaedics, Northern Health, Epping, Australia

^7^Department of Epidemiology and Preventive Medicine, Monash University, Melbourne, Victoria, Australia

^8^Department of Surgery (School of Clinical Sciences at Monash Health), Monash University

^9^Department of Cardiothoracic Surgery, Monash Health, Clayton, Australia

^10^School of Public Health, Curtin University, Perth, WA, Australia

^11^Department of Intensive Care, Royal Melbourne Hospital, Melbourne, Australia

^12^Department of Intensive Care, Austin Hospital, Melbourne, Victoria, Australia

^13^Australian and New Zealand Intensive Care Research Centre, Monash University, Melbourne, Australia

**eAppendix 1: Statistical Method – E-value Sensitivity Analysis**

This section provides a brief definition and worked examples for certain use-cases of E-values, as per VanderWeele and Ding.^1^

**Definition:** VanderWeele and Ding define the E-value as the ‘minimum strength of association on the risk ratio scale that an unmeasured confounder would need to have with both the treatment and the outcome to fully explain away a specific treatment-outcome association, conditional on the measured covariates’.^1^

**Uses and interpretation:** E-values provide a means to evaluate the possible impact of unmeasured confounders on proposed outcomes. E-values can be calculated for the following scenarios:

1. **E-values for null hypotheses**

E-values can be used to estimate the strength of an unmeasured confounder required to explain away a statistically significant outcome.

1. **E-values for non-null hypotheses**

E-values can be used to quantify confounder associations that would be needed to shift confidence intervals to exclude the null, such that outcomes are clinically significant.

## S1 Figure: Main cohort missing data prior to multiple imputation


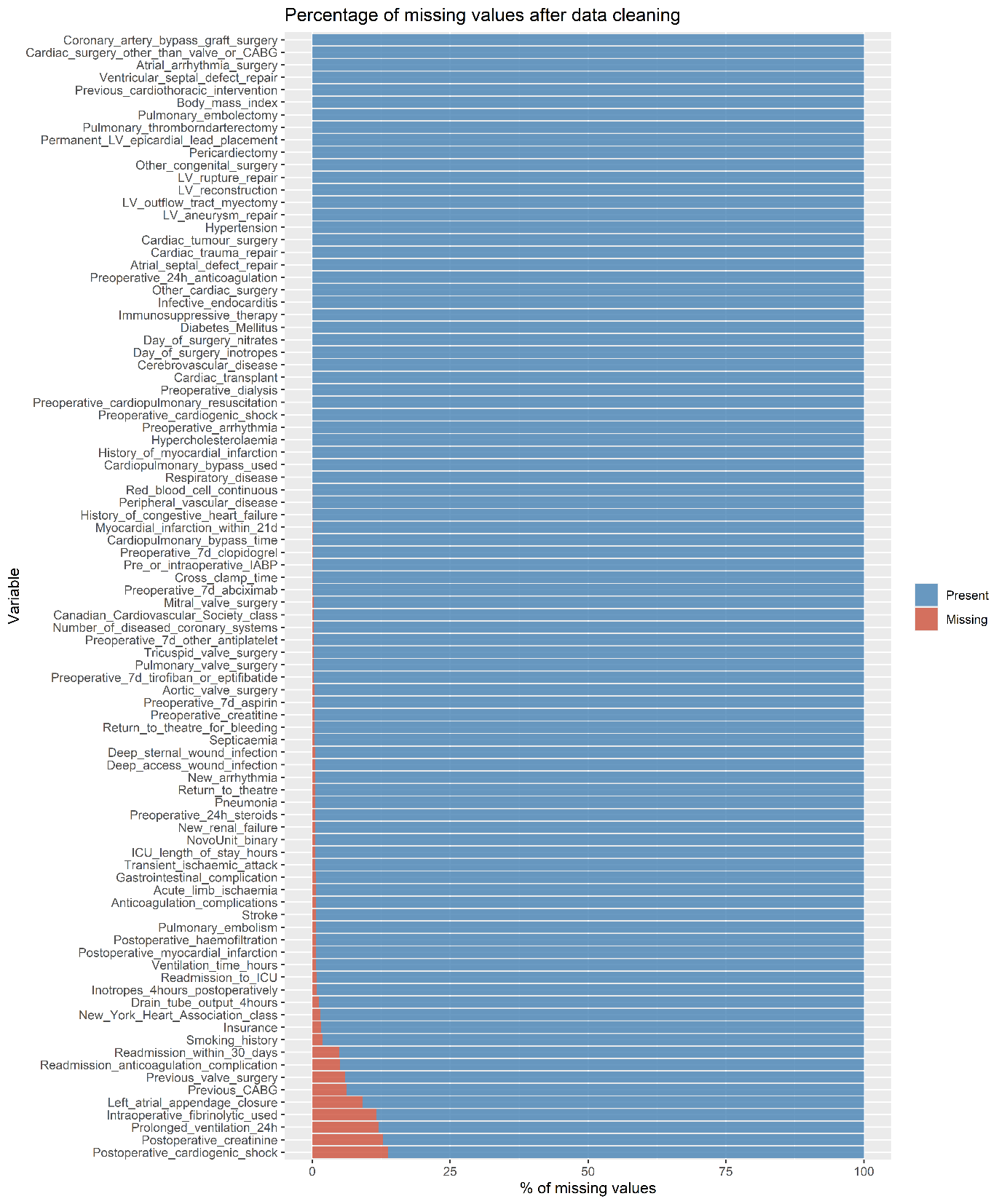


Abbreviations: PLT, platelet; CABG, coronary artery bypass graft surgery; LV, left ventricle; BMI, body mass index; ICU, intensive care unit; d, days; h, hours; IABP, intra-aortic balloon pump.

**S1 Table: Data Definitions**

| **Surgical Factors** | |
| --- | --- |
| Other Cardiac Procedure | Includes all of LV aneurysm repair, acquired VSD repair, adult ASD correction, cardiac trauma, LVOT myectomy, LV rupture repair, pericardiectomy, pulmonary thrombo-endarterectomy, LV reconstruction, pulmonary embolectomy, cardiac tumour removal, cardiac transplant, cardiopulmonary transplant, other adult congenital, permanent LV epicardial lead, left atrial appendage closure, atrial arrhythmia surgery and other. |
| **Primary Outcomes** | |
| Operative Mortality | Within 30 days from date of surgery, whether discharged or not |
| **Bleeding Complications** | |
| RTT For Bleeding | For bleeding and/or tamponade |
| **Cardiac Complications** | |
| Prolonged Inotrope Use (>4 hrs) | Inotropes used to maintain cardiac output or SVR for longer than 4 hours post-operatively. Does not include routinely administered Milrinone. |
| **Fluid Balance Complications** | |
| AKI | AKI was defined as an increased serum creatinine >0.2mmol/L and a doubling or greater increase in creatinine over baseline with the condition that the patient did not require preoperative dialysis. |
| New Postoperative RRT | Acute institution of hemofiltration or dialysis as treatment for new renal failure |
| **Infection** | |
| Wound infection | Deep sternal wound infection requiring wound debridement AND either positive cultures OR treatment with antibiotics; OR,  Infection of a thoracotomy or parasternal site requiring one of the following; wound opened with excision of tissue, positive cultures, treatment with antibiotics |
| Pneumonia | Postoperative pneumonia diagnosed by a positive culture OR radiological and clinical evidence |
| Septicaemia | Defined by positive blood cultures supported by at least two of fever, elevated granulocytes, elevated CRP, elevated ESR |
| **Hospital Resource Use** | |
| Return to Theatre | Including operative procedures in ICU that would normally be performed in theatre. |
| Readmission to ICU | During the index admission, not including patients returning to ICU following a return to theatre. |
| **Continuous Outcomes** | |
| ICU Length of Stay | Time from initial ICU admission to discharge to high dependency unit (HDU), general ward, or death |
| Ventilation time (hours) | Number of hours post-operation for which the patient was invasively ventilated |
| Chest drain tube loss (mL) | Fluid loss in milliliters from the pericardial/mediastinal drains in the first 4 hours post-operation |

**S2 Table:** Main cohort covariate balance across all weighted treatment pairs

| **Variable** | Adjusted Max Difference | Adjusted Max Variance | Adjusted Max Kolmogorov Smirnov |
| --- | --- | --- | --- |
| **Epidemiology** |  |  |  |
| Age (mean (SD)) | 0.0000 | 1.0415 | 0.0107 |
| BMI (mean (SD)) | 0.0000 | 1.1498 | 0.0167 |
| Female | 0.0000 | NA | 0.0000 |
| Insurance Status |  |  |  |
| Private Health Insurance | 0.0000 | NA | 0.0000 |
| Department of Veteran Affairs | -0.0001 | NA | 0.0001 |
| Medicare | 0.0001 | NA | 0.0001 |
| Other | 0.0001 | NA | 0.0001 |
| **Cardiac Risk Factors** |  |  |  |
| Smoking | 0.0000 | NA | 0.0001 |
| Diabetes Mellitus | 0.0000 | NA | 0.0000 |
| Hypercholesterolaemia | 0.0000 | NA | 0.0000 |
| Hypertension | 0.0000 | NA | 0.0000 |
| Congestive Heart Failure | 0.0001 | NA | 0.0001 |
| Previous Cardiac Procedure |  |  |  |
| CABG | 0.0000 | NA | 0.0000 |
| Valve | 0.0000 | NA | 0.0000 |
| **Other Comorbidities** |  |  |  |
| Cerebrovascular Disease | 0.0000 | NA | 0.0000 |
| Peripheral Vascular Disease | 0.0000 | NA | 0.0000 |
| Respiratory Disease | 0.0000 | NA | 0.0000 |
| Preoperative Dialysis | 0.0000 | NA | 0.0000 |
| Last Preop Creatinine (median [IQR]) | 0.0000 | 0.8717 | 0.0414 |
| **Cardiac Status at Time of Surgery** |  |  |  |
| Myocardial Infarction |  |  |  |
| Any MI | 0.0000 | NA | 0.0000 |
| Recent MI (< 21 days) | 0.0000 | NA | 0.0000 |
| New York Heart Association Class |  |  |  |
| I | 0.0000 | NA | 0.0001 |
| II | 0.0000 | NA | 0.0000 |
| III | 0.0001 | NA | 0.0001 |
| IV | 0.0000 | NA | 0.0000 |
| Canadian Cardiovascular Society Class |  |  |  |
| No Angina | 0.0000 | NA | 0.0000 |
| I | 0.0000 | NA | 0.0000 |
| II | 0.0000 | NA | 0.0000 |
| III | 0.0000 | NA | 0.0000 |
| IV | 0.0001 | NA | 0.0001 |
| Number of Diseased Coronary Vessels |  |  |  |
| 0 | 0.0000 | NA | 0.0000 |
| 1 | 0.0000 | NA | 0.0000 |
| 2 | 0.0000 | NA | 0.0000 |
| 3 | 0.0000 | NA | 0.0000 |
| LVEF Estimate |  |  |  |
| Normal (>60%) | 0.0000 | NA | 0.0000 |
| Mild (46-60%) | 0.0000 | NA | 0.0000 |
| Moderate (30-45%) | 0.0000 | NA | 0.0000 |
| Severe (<30%) | 0.0000 | NA | 0.0000 |
| Cardiogenic Shock | 0.0000 | NA | 0.0000 |
| Arrhythmia | 0.0000 | NA | 0.0000 |
| Infective Endocarditis | 0.0000 | NA | 0.0000 |
| **Preoperative Interventions** |  |  |  |
| Immunosuppressive Therapy (< 30 days) | 0.0000 | NA | 0.0000 |
| Inotropes (< 24 hours) | 0.0000 | NA | 0.0000 |
| Nitrates (< 24 hours) | 0.0000 | NA | 0.0000 |
| Anticoagulation (< 24 hours) | 0.0000 | NA | 0.0000 |
| Steroids (< 24 hours) | 0.0000 | NA | 0.0000 |
| Aspirin (< 7 days) | 0.0000 | NA | 0.0000 |
| Clopidogrel (< 7 days) | 0.0000 | NA | 0.0000 |
| Other antiplatelets (< 7 days) | 0.0000 | NA | 0.0000 |
| Preop Resuscitation (<1 hour) | 0.0000 | NA | 0.0000 |
| **Surgical Factors** |  |  |  |
| Urgency of Surgery |  |  |  |
| Elective | 0.0000 | NA | 0.0000 |
| Urgent | 0.0000 | NA | 0.0000 |
| Emergency | 0.0000 | NA | 0.0000 |
| Salvage | 0.0000 | NA | 0.0000 |
| Type of Surgery |  |  |  |
| Isolated CABG | 0.0000 | NA | 0.0000 |
| Isolated Valve Procedure | 0.0000 | NA | 0.0000 |
| Combined CABG/Valve | 0.0000 | NA | 0.0000 |
| Other Cardiac Procedure | 0.0000 | NA | 0.0000 |
| **Intra-/Peri-operative Processes** |  |  |  |
| Cardiopulmonary Bypass |  |  |  |
| CPB Used | 0.0000 | NA | 0.0000 |
| Cumulative CPB Time (median [IQR]) | 0.0001 | 1.0461 | 0.0154 |
| Cumulative Cross Clamp Time (median [IQR]) | 0.0000 | 1.0068 | 0.0174 |
| Intra-op Antifibrinolytic Use | 0.0000 | NA | 0.0000 |
| Intra-aortic Balloon Pump | 0.0001 | NA | 0.0001 |
| PRBC |  |  |  |
| Transfused | 0.0000 | NA | 0.0001 |
| CRYO |  |  |  |
| Transfused | 0.0000 | NA | 0.0000 |
| NOVO |  |  |  |
| Transfused | 0.0000 | NA | 0.0000 |

All values taken as maximum estimates to signify the most conservative estimates possible

**Subgroup Analysis**

An isolated CABG procedure subgroup analysis was completed to investigate a more homogenous population and support the primary outcomes seen in the total cohort. Epidemiological data relating to this subgroup is provided below.

**S3 Table**: CABG Subgroup Epidemiology

| **Variable** | **Unbalanced Cohort** | | |
| --- | --- | --- | --- |
|  | PLT (n = 7,383) | FFP (n = 2,678) | P-Value |
| **Epidemiology** |  |  |  |
| Age (mean (SD)) | 66.69 (10.25) | 67.72 (10.27) | <0.001 |
| BMI (mean (SD)) | 28.54 (5.27) | 27.97 (5.43) | <0.001 |
| Female | 1276 (17.3) | 558 (20.8) | <0.001 |
| Insurance Status |  |  | <0.001 |
| Private Health Insurance | 1918 (26.0) | 561 (20.9) |  |
| Department of Veteran Affairs | 80 (1.1) | 41 (1.5) |  |
| Medicare | 5144 (69.7) | 2007 (74.9) |  |
| Other | 241 (3.3) | 69 (2.6) |  |
| **Cardiac Risk Factors** |  |  |  |
| Smoking | 4613 (62.5) | 1666 (62.2) | 0.822 |
| Diabetes Mellitus | 2624 (35.5) | 1005 (37.5) | 0.07 |
| Hypercholesterolaemia | 5900 (79.9) | 2092 (78.1) | 0.052 |
| Hypertension | 5805 (78.6) | 2165 (80.8) | 0.017 |
| Congestive Heart Failure | 910 (12.3) | 336 (12.5) | 0.792 |
| Previous Cardiac Procedure |  |  |  |
| CABG | 517 (7.0) | 145 (5.4) | 0.005 |
| Valve | 123 (1.7) | 27 (1.0) | 0.021 |
| **Other Comorbidities** |  |  |  |
| Cerebrovascular Disease | 765 (10.4) | 286 (10.7) | 0.672 |
| Peripheral Vascular Disease | 775 (10.5) | 318 (11.9) | 0.054 |
| Respiratory Disease | 915 (12.4) | 322 (12.0) | 0.642 |
| Preoperative Dialysis | 260 (3.5) | 55 (2.1) | <0.001 |
| Last Preop Creatinine (median [IQR]) | 87.00 [74.00, 104.00] | 90.00 [75.00, 109.00] | <0.001 |
| **Cardiac Status at Time of Surgery** |  |  |  |
| Myocardial Infarction |  |  |  |
| Any MI | 4316 (58.5) | 1522 (56.8) | 0.151 |
| Recent MI (< 21 days) | 1183 (16.0) | 507 (18.9) | 0.001 |
| New York Heart Association Class |  |  | 0.535 |
| I | 3330 (45.1) | 1230 (45.9) |  |
| II | 2780 (37.7) | 975 (36.4) |  |
| III | 985 (13.3) | 356 (13.3) |  |
| IV | 288 (3.9) | 117 (4.4) |  |
| Canadian Cardiovascular Society Class |  |  | <0.001 |
| No Angina | 1228 (16.6) | 409 (15.3) |  |
| I | 779 (10.6) | 294 (11.0) |  |
| II | 2243 (30.4) | 943 (35.2) |  |
| III | 1638 (22.2) | 602 (22.5) |  |
| IV | 1495 (20.2) | 430 (16.1) |  |
| Number of Diseased Coronary Vessels |  |  | 0.504 |
| 0 | 40 (0.5) | 13 (0.5) |  |
| 1 | 215 (2.9) | 72 (2.7) |  |
| 2 | 1554 (21.0) | 532 (19.9) |  |
| 3 | 5574 (75.5) | 2061 (77.0) |  |
| LVEF Estimate |  |  | 0.003 |
| Normal (>60%) | 3260 (44.2) | 1241 (46.3) |  |
| Mild (46-60%) | 2467 (33.4) | 796 (29.7) |  |
| Moderate (30-45%) | 1212 (16.4) | 448 (16.7) |  |
| Severe (<30%) | 337 (4.6) | 140 (5.2) |  |
| Cardiogenic Shock | 196 (2.7) | 58 (2.2) | 0.19 |
| Arrhythmia | 750 (10.2) | 315 (11.8) | 0.023 |
| Infective Endocarditis | 3 (0.0) | 1 (0.0) | 1 |
| **Preoperative Interventions** |  |  |  |
| Immunosuppressive Therapy (< 30 days) | 225 (3.0) | 53 (2.0) | 0.005 |
| Inotropes (< 24 hours) | 185 (2.5) | 67 (2.5) | 1 |
| Nitrates (< 24 hours) | 590 (8.0) | 146 (5.5) | <0.001 |
| Anticoagulation (< 24 hours) | 2216 (30.0) | 681 (25.4) | <0.001 |
| Steroids (< 24 hours) | 128 (1.7) | 50 (1.9) | 0.717 |
| Aspirin (< 7 days) | 6336 (85.8) | 2029 (75.8) | <0.001 |
| Clopidogrel (< 7 days) | 1709 (23.1) | 403 (15.0) | <0.001 |
| Other antiplatelets (< 7 days) | 571 (7.7) | 178 (6.6) | 0.073 |
| Preop Resuscitation (<1 hour) | 96 (1.3) | 24 (0.9) | 0.122 |
| **Surgical Factors** |  |  |  |
| Urgency of Surgery |  |  | <0.001 |
| Elective | 3559 (48.2) | 1498 (55.9) |  |
| Urgent | 3179 (43.1) | 1063 (39.7) |  |
| Emergency | 616 (8.3) | 111 (4.1) |  |
| Salvage | 29 (0.4) | 6 (0.2) |  |
| Type of Surgery |  |  | 0.000 |
| Isolated CABG | 7383 (100.0) | 2678 (100.0) |  |
| Isolated Valve Procedure | 0 | 0 |  |
| Combined CABG/Valve | 0 | 0 |  |
| Other Cardiac Procedure | 0 | 0 |  |
| **Intra-/Peri-operative Processes** |  |  |  |
| Cardiopulmonary Bypass |  |  |  |
| CPB Used | 7034 (95.3) | 2497 (93.2) | <0.001 |
| Cumulative CPB Time (median [IQR]) | 89.00 [67.00, 113.00] | 92.00 [69.00, 117.00] | 0.014 |
| Cumulative Cross Clamp Time (median [IQR]) | 62.00 [44.00, 83.00] | 62.00 [44.00, 86.00] | 0.676 |
| Intra-op Antifibrinolytic Use | 6223 (84.3) | 2290 (85.5) | 0.141 |
| Intra-aortic Balloon Pump | 676 (9.2) | 157 (5.9) | <0.001 |
| PRBC |  |  |  |
| Transfused | 4169 (56.5) | 1801 (67.3) | <0.001 |
| Cumulative Units (median [IQR]) | 1.00 [0.00, 3.00] | 2.00 [0.00, 3.00] | <0.001 |
| CRYO |  |  |  |
| Transfused | 1360 (18.4) | 383 (14.3) | <0.001 |
| Cumulative Units (median [IQR]) | 0.00 [0.00, 0.00] | 0.00 [0.00, 0.00] | <0.001 |
| FFP |  |  |  |
| Transfused | 0 (0.0) | 2678 (100.0) | <0.001 |
| Cumulative Units (median [IQR]) | 0.00 [0.00, 0.00] | 2.00 [2.00, 4.00] | <0.001 |
| PLTS |  |  |  |
| Transfused | 7383 (100.0) | 0 (0.0) | <0.001 |
| Cumulative Units (median [IQR]) | 1.00 [1.00, 3.00] | 0.00 [0.00, 0.00] | <0.001 |
| NOVO |  |  |  |
| Transfused | 16 (0.2) | 3 (0.1) | 0.418 |
| Cumulative Units (median [IQR]) | 0.00 [0.00, 0.00] | 0.00 [0.00, 0.00] | 0.285 |

Primary outcomes for this subgroup analysis are presented in the Table below

**S4 Table**: CABG Subgroup Outcomes

| **Outcomes** | **RR** | **CI (95%)** | **P-Value** |
| --- | --- | --- | --- |
| **Primary Outcomes** |  |  |  |
| Operative Mortality | 1.71 | (1.28,2.27) | <0.001 |
| **Bleeding Complications** |  |  |  |
| Return to Theatre | 1.04 | (0.89,1.21) | 0.675 |
| Return to Theatre for Bleeding | 0.72 | (0.57,0.92) | 0.020 |
| **Cardiac Complications** |  |  |  |
| Prolonged Inotrope Use (>4 hrs) | 0.91 | (0.87,0.95) | <0.001 |
| **Fluid Balance Complications** |  |  |  |
| AKI | 1.11 | (0.90,1.38) | 0.033 |
| New Postoperative RRT | 1.34 | (0.98,1.82) | 0.020 |
| **Infection** |  |  |  |
| All Infection | 0.94 | (0.79,1.11) | 0.062 |
| Pneumonia | 0.80 | (0.66,0.97) | 0.004 |
| Septicaemia | 1.20 | (0.78,1.86) | 0.866 |
| Wound Infection | 1.88 | (1.19,2.99) | 0.014 |
| **Hospital Resource Use** |  |  |  |
| Readmission to ICU | 1.18 | (0.94,1.49) | 0.001 |
| **Continuous Outcomes** | **AMD** | **CI (95%)** | **P-Value** |
| ICU Length of Stay, hrs | -0.12 | (-6.64,6.40) | 0.193 |
| Ventilation Time, hrs | -0.05 | (-4.63,4.53) | 0.982 |
| 4hr Chest Drain Output, ml | 22.70 | (9.93,35.46) | <0.001 |

**S5 Table**: CABG Subgroup Balance Summary

| **Variable** | Adjusted Max Difference | Adjusted Max Variance | Adjusted Max Kolmogorov Smirnov |
| --- | --- | --- | --- |
| **Epidemiology** |  |  |  |
| Age (mean (SD)) | 0.0000 | 1.0163 | 0.0154 |
| BMI (mean (SD)) | 0.0000 | 1.2137 | 0.0204 |
| Female | 0.0001 | NA | 0.0001 |
| Insurance Status |  |  |  |
| Private Health Insurance | 0.0000 | NA | 0.0001 |
| Department of Veteran Affairs | 0.0000 | NA | 0.0000 |
| Medicare | 0.0000 | NA | 0.0001 |
| Other | 0.0000 | NA | 0.0000 |
| **Cardiac Risk Factors** |  |  |  |
| Smoking | 0.0000 | NA | 0.0001 |
| Diabetes Mellitus | 0.0000 | NA | 0.0001 |
| Hypercholesterolaemia | 0.0001 | NA | 0.0001 |
| Hypertension | 0.0000 | NA | 0.0000 |
| Congestive Heart Failure | 0.0000 | NA | 0.0000 |
| Previous Cardiac Procedure |  |  |  |
| CABG | 0.0000 | NA | 0.0000 |
| Valve | 0.0000 | NA | 0.0000 |
| **Other Comorbidities** |  |  |  |
| Cerebrovascular Disease | 0.0000 | NA | 0.0000 |
| Peripheral Vascular Disease | 0.0000 | NA | 0.0000 |
| Respiratory Disease | 0.0000 | NA | 0.0000 |
| Preoperative Dialysis | 0.0000 | NA | 0.0000 |
| Last Preop Creatinine (median [IQR]) | 0.0000 | 0.8309 | 0.0438 |
| **Cardiac Status at Time of Surgery** |  |  |  |
| Myocardial Infarction |  |  |  |
| Any MI | 0.0000 | NA | 0.0000 |
| Recent MI (< 21 days) | 0.0000 | NA | 0.0001 |
| New York Heart Association Class |  |  |  |
| I | 0.0000 | NA | 0.0000 |
| II | 0.0000 | NA | 0.0000 |
| III | 0.0000 | NA | 0.0000 |
| IV | 0.0000 | NA | 0.0000 |
| Canadian Cardiovascular Society Class |  |  |  |
| No Angina | 0.0000 | NA | 0.0001 |
| I | 0.0000 | NA | 0.0000 |
| II | 0.0000 | NA | 0.0001 |
| III | 0.0000 | NA | 0.0000 |
| IV | 0.0001 | NA | 0.0001 |
| Number of Diseased Coronary Vessels |  |  |  |
| 0 | 0.0000 | NA | 0.0000 |
| 1 | 0.0000 | NA | 0.0001 |
| 2 | 0.0001 | NA | 0.0001 |
| 3 | 0.0000 | NA | 0.0001 |
| LVEF Estimate |  |  |  |
| Normal (>60%) | 0.0001 | NA | 0.0001 |
| Mild (46-60%) | 0.0000 | NA | 0.0001 |
| Moderate (30-45%) | 0.0000 | NA | 0.0000 |
| Severe (<30%) | 0.0000 | NA | 0.0000 |
| Cardiogenic Shock | 0.0000 | NA | 0.0000 |
| Arrhythmia | 0.0000 | NA | 0.0000 |
| Infective Endocarditis | 0.0000 | NA | 0.0000 |
| **Preoperative Interventions** |  |  |  |
| Immunosuppressive Therapy (< 30 days) | 0.0000 | NA | 0.0000 |
| Inotropes (< 24 hours) | 0.0000 | NA | 0.0000 |
| Nitrates (< 24 hours) | 0.0000 | NA | 0.0001 |
| Anticoagulation (< 24 hours) | 0.0000 | NA | 0.0000 |
| Steroids (< 24 hours) | 0.0000 | NA | 0.0000 |
| Aspirin (< 7 days) | 0.0000 | NA | 0.0000 |
| Clopidogrel (< 7 days) | 0.0000 | NA | 0.0000 |
| Other antiplatelets (< 7 days) | 0.0000 | NA | 0.0000 |
| Preop Resuscitation (<1 hour) | 0.0000 | NA | 0.0000 |
| **Surgical Factors** |  |  |  |
| Urgency of Surgery |  |  |  |
| Elective | 0.0000 | NA | 0.0001 |
| Urgent | 0.0001 | NA | 0.0001 |
| Emergency | 0.0000 | NA | 0.0000 |
| Salvage | 0.0000 | NA | 0.0000 |
| Type of Surgery |  |  |  |
| Isolated CABG | 0.0000 | 0.0000 | 0.0000 |
| Isolated Valve Procedure | 0.0000 | 0.0000 | 0.0000 |
| Combined CABG/Valve | 0.0000 | 0.0000 | 0.0000 |
| Other Cardiac Procedure | 0.0000 | 0.0000 | 0.0000 |
| **Intra-/Peri-operative Processes** |  |  |  |
| Cardiopulmonary Bypass |  |  |  |
| CPB Used | 0.0000 | NA | 0.0000 |
| Cumulative CPB Time (median [IQR]) | 0.0000 | 0.7934 | 0.0170 |
| Cumulative Cross Clamp Time (median [IQR]) | 0.0000 | 1.0519 | 0.0246 |
| Intra-op Antifibrinolytic Use | 0.0000 | NA | 0.0001 |
| Intra-aortic Balloon Pump | 0.0001 | NA | 0.0001 |
| PRBC |  |  |  |
| Transfused | 0.0001 | NA | 0.0001 |
| CRYO |  |  |  |
| Transfused | 0.0000 | NA | 0.0000 |
| NOVO |  |  |  |
| Transfused | 0.0000 | NA | 0.0000 |

A subgroup analysis on the most recent 5 years (2017-2021) of patient encounters was completed to investigate any recency bias and support the primary outcomes seen in the total cohort. Epidemiological data relating to this subgroup is provided below.

**S6 Table**: 5-year Subgroup Epidemiology

| **Variable** | **Unbalanced Cohort** | | |
| --- | --- | --- | --- |
|  | PLT (n = 9,039) | FFP (n = 1,771) | P-Value |
| **Epidemiology** |  |  |  |
| Age (mean (SD)) | 66.06 (12.59) | 66.01 (12.67) | 0.879 |
| BMI (mean (SD)) | 28.34 (5.61) | 28.05 (6.07) | 0.053 |
| Female | 2043 (22.6) | 451 (25.5) | 0.01 |
| Insurance Status |  |  | <0.001 |
| Private Health Insurance | 2564 (28.4) | 472 (26.7) |  |
| Department of Veteran Affairs | 80 (0.9) | 13 (0.7) |  |
| Medicare | 5888 (65.1) | 1233 (69.6) |  |
| Other | 507 (5.6) | 53 (3.0) |  |
| **Cardiac Risk Factors** |  |  |  |
| Smoking | 4942 (54.7) | 904 (51.0) | 0.005 |
| Diabetes Mellitus | 2490 (27.5) | 544 (30.7) | 0.007 |
| Hypercholesterolaemia | 5859 (64.8) | 1080 (61.0) | 0.002 |
| Hypertension | 6254 (69.2) | 1257 (71.0) | 0.143 |
| Congestive Heart Failure | 1787 (19.8) | 438 (24.7) | <0.001 |
| Previous Cardiac Procedure |  |  |  |
| CABG | 822 (9.1) | 140 (7.9) | 0.119 |
| Valve | 859 (9.5) | 134 (7.6) | 0.011 |
| **Other Comorbidities** |  |  |  |
| Cerebrovascular Disease | 987 (10.9) | 189 (10.7) | 0.792 |
| Peripheral Vascular Disease | 742 (8.2) | 144 (8.1) | 0.951 |
| Respiratory Disease | 1372 (15.2) | 277 (15.6) | 0.647 |
| Preoperative Dialysis | 294 (3.3) | 39 (2.2) | 0.024 |
| Last Preop Creatinine (median [IQR]) | 87.00 [74.00, 106.00] | 89.00 [74.00, 110.00] | 0.104 |
| **Cardiac Status at Time of Surgery** |  |  |  |
| Myocardial Infarction |  |  |  |
| Any MI | 3249 (35.9) | 590 (33.3) | 0.037 |
| Recent MI (< 21 days) | 1040 (11.5) | 203 (11.5) | 0.991 |
| New York Heart Association Class |  |  | 0.012 |
| I | 3219 (35.6) | 581 (32.8) |  |
| II | 3448 (38.1) | 676 (38.2) |  |
| III | 1796 (19.9) | 370 (20.9) |  |
| IV | 576 (6.4) | 144 (8.1) |  |
| Canadian Cardiovascular Society Class |  |  | <0.001 |
| No Angina | 4345 (48.1) | 914 (51.6) |  |
| I | 917 (10.1) | 202 (11.4) |  |
| II | 1789 (19.8) | 375 (21.2) |  |
| III | 1026 (11.4) | 162 (9.1) |  |
| IV | 962 (10.6) | 118 (6.7) |  |
| Number of Diseased Coronary Vessels |  |  | 0.010 |
| 0 | 3066 (33.9) | 672 (37.9) |  |
| 1 | 739 (8.2) | 126 (7.1) |  |
| 2 | 1337 (14.8) | 243 (13.7) |  |
| 3 | 3897 (43.1) | 730 (41.2) |  |
| LVEF Estimate |  |  | <0.001 |
| Normal (>60%) | 3867 (42.8) | 813 (45.9) |  |
| Mild (46-60%) | 3143 (34.8) | 551 (31.1) |  |
| Moderate (30-45%) | 1376 (15.2) | 242 (13.7) |  |
| Severe (<30%) | 459 (5.1) | 138 (7.8) |  |
| Cardiogenic Shock | 306 (3.4) | 66 (3.7) | 0.516 |
| Arrhythmia | 1915 (21.2) | 458 (25.9) | <0.001 |
| Infective Endocarditis | 471 (5.2) | 120 (6.8) | 0.01 |
| **Preoperative Interventions** |  |  |  |
| Immunosuppressive Therapy (< 30 days) | 341 (3.8) | 53 (3.0) | 0.125 |
| Inotropes (< 24 hours) | 363 (4.0) | 108 (6.1) | <0.001 |
| Nitrates (< 24 hours) | 396 (4.4) | 55 (3.1) | 0.017 |
| Anticoagulation (< 24 hours) | 2425 (26.8) | 470 (26.5) | 0.824 |
| Steroids (< 24 hours) | 279 (3.1) | 45 (2.5) | 0.248 |
| Aspirin (< 7 days) | 5748 (63.6) | 974 (55.0) | <0.001 |
| Clopidogrel (< 7 days) | 1022 (11.3) | 97 (5.5) | <0.001 |
| Other antiplatelets (< 7 days) | 489 (5.4) | 102 (5.8) | 0.593 |
| Preop Resuscitation (<1 hour) | 96 (1.1) | 18 (1.0) | 0.964 |
| **Surgical Factors** |  |  |  |
| Urgency of Surgery |  |  | <0.001 |
| Elective | 5066 (56.0) | 1051 (59.3) |  |
| Urgent | 3150 (34.8) | 626 (35.3) |  |
| Emergency | 789 (8.7) | 86 (4.9) |  |
| Salvage | 34 (0.4) | 8 (0.5) |  |
| Type of Surgery |  |  | 0.007 |
| Isolated CABG | 3913 (43.3) | 717 (40.5) |  |
| Isolated Valve Procedure | 1808 (20.0) | 363 (20.5) |  |
| Combined CABG/Valve | 1568 (17.3) | 289 (16.3) |  |
| Other Cardiac Procedure | 1750 (19.4) | 402 (22.7) |  |
| **Intra-/Peri-operative Processes** |  |  |  |
| Cardiopulmonary Bypass |  |  |  |
| CPB Used | 8811 (97.5) | 1737 (98.1) | 0.155 |
| Cumulative CPB Time (median [IQR]) | 109.00 [78.00, 152.00] | 108.00 [78.00, 142.50] | 0.076 |
| Cumulative Cross Clamp Time (median [IQR]) | 76.00 [51.00, 109.00] | 76.00 [50.00, 104.00] | 0.23 |
| Intra-op Antifibrinolytic Use | 8150 (90.2) | 1622 (91.6) | 0.07 |
| Intra-aortic Balloon Pump | 535 (5.9) | 65 (3.7) | <0.001 |
| PRBC |  |  |  |
| Transfused | 5288 (58.5) | 1096 (61.9) | 0.009 |
| Cumulative Units (median [IQR]) | 1.00 [0.00, 3.00] | 1.00 [0.00, 3.00] | 0.002 |
| CRYO |  |  |  |
| Transfused | 3153 (34.9) | 467 (26.4) | <0.001 |
| Cumulative Units (median [IQR]) | 0.00 [0.00, 5.00] | 0.00 [0.00, 1.00] | <0.001 |
| FFP |  |  |  |
| Transfused | 0 (0.0) | 1771 (100.0) | <0.001 |
| Cumulative Units (median [IQR]) | 0.00 [0.00, 0.00] | 2.00 [2.00, 3.00] | <0.001 |
| PLTS |  |  |  |
| Transfused | 9039 (100.0) | 0 (0.0) | <0.001 |
| Cumulative Units (median [IQR]) | 1.00 [1.00, 3.00] | 0.00 [0.00, 0.00] | <0.001 |
| NOVO |  |  |  |
| Transfused | 62 (0.7) | 8 (0.5) | 0.336 |
| Cumulative Units (median [IQR]) | 0.00 [0.00, 0.00] | 0.00 [0.00, 0.00] | 0.261 |

Primary outcomes for this subgroup analysis are presented in the Table below

**S7 Table**: 5-year Subgroup Outcomes

| **Outcomes** | **RR** | **CI (95%)** | **P-Value** |
| --- | --- | --- | --- |
| **Primary Outcomes** |  |  |  |
| Operative Mortality | 1.59 | (1.20,2.10) | <0.001 |
| **Bleeding Complications** |  |  |  |
| Return to Theatre | 0.99 | (0.87,1.14) | 0.675 |
| Return to Theatre for Bleeding | 0.75 | (0.59,0.95) | 0.020 |
| **Cardiac Complications** |  |  |  |
| Prolonged Inotrope Use (>4 hrs) | 0.90 | (0.87,0.95) | <0.001 |
| **Fluid Balance Complications** |  |  |  |
| AKI | 1.31 | (1.09,1.58) | 0.033 |
| New Postoperative RRT | 1.45 | (1.13,1.86) | 0.020 |
| **Infection** |  |  |  |
| All Infection | 0.96 | (0.80,1.14) | 0.062 |
| Pneumonia | 0.98 | (0.80,1.21) | 0.004 |
| Septicaemia | 1.03 | (0.67,1.57) | 0.866 |
| Wound Infection | 1.19 | (0.69,2.05) | 0.014 |
| **Hospital Resource Use** |  |  |  |
| Readmission to ICU | 1.14 | (0.91,1.44) | 0.001 |
| **Continuous Outcomes** |  |  |  |
| ICU Length of Stay, hrs | 13.75 | (6.03,21.48) | 0.193 |
| Ventilation Time, hrs | 1.84 | (-2.42,6.10) | 0.396 |
| 4hr Chest Drain Output, ml | 16.04 | (2.43,29.64) | 0.021 |

**S8 Table**: 5-year Subgroup Balance Summary

| **Variable** | Adjusted Max Difference | Adjusted Max Variance | Adjusted Max Kolmogorov Smirnov |
| --- | --- | --- | --- |
| **Epidemiology** |  |  |  |
| Age (mean (SD)) | 0.0001 | 0.9706 | 0.0190 |
| BMI (mean (SD)) | 0.0001 | 1.1813 | 0.0227 |
| Female | 0.0000 | NA | 0.0000 |
| Insurance Status |  |  |  |
| Private Health Insurance | 0.0000 | NA | 0.0000 |
| Department of Veteran Affairs | 0.0000 | NA | 0.0000 |
| Medicare | 0.0000 | NA | 0.0000 |
| Other | 0.0000 | NA | 0.0000 |
| **Cardiac Risk Factors** |  |  |  |
| Smoking | 0.0001 | NA | 0.0001 |
| Diabetes Mellitus | 0.0000 | NA | 0.0000 |
| Hypercholesterolaemia | 0.0000 | NA | 0.0000 |
| Hypertension | 0.0000 | NA | 0.0000 |
| Congestive Heart Failure | 0.0000 | NA | 0.0000 |
| Previous Cardiac Procedure |  |  |  |
| CABG | 0.0000 | NA | 0.0000 |
| Valve | 0.0000 | NA | 0.0000 |
| **Other Comorbidities** |  |  |  |
| Cerebrovascular Disease | 0.0000 | NA | 0.0000 |
| Peripheral Vascular Disease | 0.0000 | NA | 0.0000 |
| Respiratory Disease | 0.0000 | NA | 0.0000 |
| Preoperative Dialysis | 0.0000 | NA | 0.0000 |
| Last Preop Creatinine (median [IQR]) | 0.0000 | 0.8921 | 0.0271 |
| **Cardiac Status at Time of Surgery** |  |  |  |
| Myocardial Infarction |  |  |  |
| Any MI | 0.0000 | NA | 0.0000 |
| Recent MI (< 21 days) | 0.0000 | NA | 0.0000 |
| New York Heart Association Class |  |  |  |
| I | 0.0000 | NA | 0.0000 |
| II | -0.0001 | NA | 0.0001 |
| III | 0.0000 | NA | 0.0000 |
| IV | 0.0000 | NA | 0.0000 |
| Canadian Cardiovascular Society Class |  |  |  |
| No Angina | 0.0000 | NA | 0.0000 |
| I | 0.0000 | NA | 0.0000 |
| II | 0.0000 | NA | 0.0000 |
| III | 0.0000 | NA | 0.0000 |
| IV | 0.0000 | NA | 0.0000 |
| Number of Diseased Coronary Vessels |  |  |  |
| 0 | 0.0000 | NA | 0.0000 |
| 1 | 0.0000 | NA | 0.0000 |
| 2 | 0.0000 | NA | 0.0001 |
| 3 | 0.0000 | NA | 0.0000 |
| LVEF Estimate |  |  |  |
| Normal (>60%) | 0.0001 | NA | 0.0001 |
| Mild (46-60%) | 0.0000 | NA | 0.0000 |
| Moderate (30-45%) | 0.0000 | NA | 0.0000 |
| Severe (<30%) | 0.0000 | NA | 0.0000 |
| Cardiogenic Shock | 0.0000 | NA | 0.0000 |
| Arrhythmia | 0.0000 | NA | 0.0000 |
| Infective Endocarditis | 0.0000 | NA | 0.0000 |
| **Preoperative Interventions** |  |  |  |
| Immunosuppressive Therapy (< 30 days) | 0.0000 | NA | 0.0000 |
| Inotropes (< 24 hours) | 0.0000 | NA | 0.0000 |
| Nitrates (< 24 hours) | 0.0000 | NA | 0.0000 |
| Anticoagulation (< 24 hours) | 0.0000 | NA | 0.0000 |
| Steroids (< 24 hours) | 0.0000 | NA | 0.0000 |
| Aspirin (< 7 days) | 0.0000 | NA | 0.0000 |
| Clopidogrel (< 7 days) | 0.0000 | NA | 0.0000 |
| Other antiplatelets (< 7 days) | 0.0000 | NA | 0.0000 |
| Preop Resuscitation (<1 hour) | -0.0001 | NA | 0.0001 |
| **Surgical Factors** |  |  |  |
| Urgency of Surgery |  |  |  |
| Elective | 0.0000 | NA | 0.0000 |
| Urgent | 0.0000 | NA | 0.0000 |
| Emergency | 0.0000 | NA | 0.0001 |
| Salvage | 0.0001 | NA | 0.0001 |
| Type of Surgery |  |  |  |
| Isolated CABG | 0.0000 | NA | 0.0000 |
| Isolated Valve Procedure | 0.0000 | NA | 0.0000 |
| Combined CABG/Valve | 0.0000 | NA | 0.0000 |
| Other Cardiac Procedure | 0.0000 | NA | 0.0000 |
| **Intra-/Peri-operative Processes** |  |  |  |
| Cardiopulmonary Bypass |  |  |  |
| CPB Used | 0.0000 | NA | 0.0000 |
| Cumulative CPB Time (median [IQR]) | 0.0001 | 1.2128 | 0.0282 |
| Cumulative Cross Clamp Time (median [IQR]) | 0.0000 | 0.9934 | 0.0222 |
| Intra-op Antifibrinolytic Use | 0.0000 | NA | 0.0000 |
| Intra-aortic Balloon Pump | 0.0000 | NA | 0.0000 |
| PRBC |  |  |  |
| Transfused | 0.0001 | NA | 0.0001 |
| CRYO |  |  |  |
| Transfused | 0.0000 | NA | 0.0000 |
| NOVO |  |  |  |
| Transfused | 0.0000 | NA | 0.0001 |

A subgroup analysis on patients not exposed to cryoprecipitate was completed to investigate any bias from transfusion with other plasma-derived blood products. Epidemiological data relating to this subgroup is provided below.

**S9 Table**: No Cryoprecipitate Subgroup Epidemiology

| **Variable** | **Unbalanced Cohort** | | |
| --- | --- | --- | --- |
|  | PLT (n = 11,235) | FFP (n = 5,161) | P-Value |
| **Epidemiology** |  |  |  |
| Age (mean (SD)) | 66.99 (11.97) | 67.02 (13.08) | 0.901 |
| BMI (mean (SD)) | 28.43 (5.45) | 27.70 (5.63) | <0.001 |
| Female | 2612 (23.2) | 1514 (29.3) | <0.001 |
| Insurance Status |  |  | <0.001 |
| Private Health Insurance | 3154 (28.1) | 1233 (23.9) |  |
| Department of Veteran Affairs | 139 (1.2) | 80 (1.6) |  |
| Medicare | 7523 (67.0) | 3724 (72.2) |  |
| Other | 419 (3.7) | 124 (2.4) |  |
| **Cardiac Risk Factors** |  |  |  |
| Smoking | 6485 (57.7) | 2859 (55.4) | 0.006 |
| Diabetes Mellitus | 3350 (29.8) | 1488 (28.8) | 0.205 |
| Hypercholesterolaemia | 7851 (69.9) | 3256 (63.1) | <0.001 |
| Hypertension | 8303 (73.9) | 3671 (71.1) | <0.001 |
| Congestive Heart Failure | 2402 (21.4) | 1520 (29.5) | <0.001 |
| Previous Cardiac Procedure |  |  |  |
| CABG | 759 (6.8) | 285 (5.5) | 0.003 |
| Valve | 675 (6.0) | 278 (5.4) | 0.123 |
| **Other Comorbidities** |  |  |  |
| Cerebrovascular Disease | 1315 (11.7) | 613 (11.9) | 0.769 |
| Peripheral Vascular Disease | 1083 (9.6) | 512 (9.9) | 0.592 |
| Respiratory Disease | 1612 (14.3) | 770 (14.9) | 0.347 |
| Preoperative Dialysis | 385 (3.4) | 103 (2.0) | <0.001 |
| Last Preop Creatinine (median [IQR]) | 88.00 [74.00,108.00] | 90.00 [75.00, 111.00] | 0.001 |
| **Cardiac Status at Time of Surgery** |  |  |  |
| Myocardial Infarction |  |  |  |
| Any MI | 4744 (42.2) | 1878 (36.4) | <0.001 |
| Recent MI (< 21 days) | 1548 (13.8) | 761 (14.7) | 0.103 |
| New York Heart Association Class |  |  | <0.001 |
| I | 4067 (36.2) | 1681 (32.6) |  |
| II | 4256 (37.9) | 1821 (35.3) |  |
| III | 2271 (20.2) | 1196 (23.2) |  |
| IV | 641 (5.7) | 463 (9.0) |  |
| Canadian Cardiovascular Society Class |  |  | <0.001 |
| No Angina | 4315 (38.4) | 2200 (42.6) |  |
| I | 1139 (10.1) | 516 (10.0) |  |
| II | 2570 (22.9) | 1224 (23.7) |  |
| III | 1724 (15.3) | 729 (14.1) |  |
| IV | 1487 (13.2) | 492 (9.5) |  |
| Number of Diseased Coronary Vessels |  |  | <0.001 |
| 0 | 2831 (25.2) | 1709 (33.1) |  |
| 1 | 898 (8.0) | 404 (7.8) |  |
| 2 | 1939 (17.3) | 760 (14.7) |  |
| 3 | 5567 (49.6) | 2288 (44.3) |  |
| LVEF Estimate < 30% | 1238 (11.0) | 677 (13.1) | <0.001 |
| Cardiogenic Shock | 340 (3.0) | 166 (3.2) | 0.545 |
| Arrhythmia | 2145 (19.1) | 1315 (25.5) | <0.001 |
| Infective Endocarditis | 421 (3.7) | 268 (5.2) | <0.001 |
| **Preoperative Interventions** |  |  |  |
| Immunosuppressive Therapy (< 30 days) | 384 (3.4) | 134 (2.6) | 0.006 |
| Inotropes (< 24 hours) | 348 (3.1) | 247 (4.8) | <0.001 |
| Nitrates (< 24 hours) | 587 (5.2) | 193 (3.7) | <0.001 |
| Anticoagulation (< 24 hours) | 2828 (25.2) | 1179 (22.8) | 0.001 |
| Steroids (< 24 hours) | 282 (2.5) | 119 (2.3) | 0.464 |
| Aspirin (< 7 days) | 7587 (67.5) | 2692 (52.2) | <0.001 |
| Clopidogrel (< 7 days) | 1763 (15.7) | 456 (8.8) | <0.001 |
| Other antiplatelets (< 7 days) | 752 (6.7) | 296 (5.7) | 0.022 |
| Preop Resuscitation (<1 hour) | 122 (1.1) | 57 (1.1) | 0.980 |
| **Surgical Factors** |  |  |  |
| Urgency of Surgery |  |  | <0.001 |
| Elective | 6574 (58.5) | 3262 (63.2) |  |
| Urgent | 3843 (34.2) | 1640 (31.8) |  |
| Emergency | 788 (7.0) | 243 (4.7) |  |
| Salvage | 30 (0.3) | 16 (0.3) |  |
| Type of Surgery |  |  | <0.001 |
| Isolated CABG | 6023 (53.6) | 2295 (44.5) |  |
| Isolated Valve Procedure | 1855 (16.5) | 1008 (19.5) |  |
| Combined CABG/Valve | 1873 (16.7) | 941 (18.2) |  |
| Other Cardiac Procedure | 1484 (13.2) | 917 (17.8) |  |
| **Intra-/Peri-operative Processes** |  |  |  |
| Cardiopulmonary Bypass |  |  |  |
| CPB Used | 10887 (96.9) | 4969 (96.3) | 0.043 |
| Cumulative CPB Time (median [IQR]) | 102.00 [76.00, 138.00] | 105.00 [79.00, 131.00] | 0.001 |
| Cumulative Cross Clamp Time (median [IQR]) | 73.00 [51.00, 103.00] | 75.00 [51.00, 105.00] | 0.286 |
| Intra-op Antifibrinolytic Use | 9550 (85.0) | 4377 (84.8) | 0.766 |
| Intra-aortic Balloon Pump | 804 (7.2) | 261 (5.1) | <0.001 |
| PRBC |  |  |  |
| Transfused | 6338 (56.4) | 3458 (67.0) | <0.001 |
| Cumulative Units (median [IQR]) | 1.00 [0.00, 3.00] | 2.00 [0.00, 4.00] | <0.001 |
| CRYO |  |  |  |
| Transfused | 0 (0.0) | 0 (0.0) | NA |
| Cumulative Units (median [IQR]) | 0.00 [0.00, 0.00] | 0.00 [0.00, 0.00] | NA |
| FFP |  |  |  |
| Transfused | 0 (0.0) | 5161 (100.0) | <0.001 |
| Cumulative Units (median [IQR]) | 0.00 [0.00, 0.00] | 2.00 [2.00, 4.00] | <0.001 |
| PLTS |  |  |  |
| Transfused | 11235 (100.0) | 0 (0.0) | <0.001 |
| Cumulative Units (median [IQR]) | 1.00 [1.00, 2.00] | 0.00 [0.00, 0.00] | <0.001 |
| NOVO |  |  |  |
| Transfused | 36 (0.3) | 13 (0.3) | 0.553 |
| Cumulative Units (median [IQR]) | 0.00 [0.00, 0.00] | 0.00 [0.00, 0.00] | 0.454 |

Primary outcomes for this subgroup analysis are presented in the Table below

**S10 Table**: No Cryoprecipitate Subgroup Outcomes

| **Outcomes** | **RR** | **CI (95%)** | **P-Value** |
| --- | --- | --- | --- |
| **Primary Outcomes** |  |  |  |
| Operative Mortality | 1.62 | (1.36,1.94) | <0.001 |
| **Bleeding Complications** |  |  |  |
| Return to Theatre | 1.05 | (0.95,1.16) | 0.675 |
| Return to Theatre for Bleeding | 0.89 | (0.75,1.06) | 0.020 |
| **Cardiac Complications** |  |  |  |
| Prolonged Inotrope Use (>4 hrs) | 0.93 | (0.91,0.96) | <0.001 |
| **Fluid Balance Complications** |  |  |  |
| AKI | 1.14 | (1.01,1.30) | 0.033 |
| New Postoperative RRT | 1.21 | (1.01,1.44) | 0.020 |
| **Infection** |  |  |  |
| All Infection | 1.00 | (0.89,1.12) | 0.062 |
| Pneumonia | 0.92 | (0.81,1.05) | 0.004 |
| Septicaemia | 1.10 | (0.86,1.42) | 0.866 |
| Wound Infection | 1.71 | (1.23,2.39) | 0.014 |
| **Hospital Resource Use** |  |  |  |
| Readmission to ICU | 1.34 | (1.16,1.55) | 0.001 |
| **Continuous Outcomes** |  |  |  |
| ICU Length of Stay, hrs | 3.52 | (-1.40,8.45) | 0.193 |
| Ventilation Time, hrs | 1.84 | (-2.42,6.10) | 0.396 |
| 4hr Chest Drain Output, ml | 16.04 | (2.43,29.64) | 0.021 |

**S11 Table**: No Cryoprecipitate Subgroup Balance Summary

| **Variable** | Adjusted Max Difference | Adjusted Max Variance | Adjusted Max Kolmogorov Smirnov |
| --- | --- | --- | --- |
| **Epidemiology** |  |  |  |
| Age (mean (SD)) | 0.0000 | 1.0239 | 0.0144 |
| BMI (mean (SD)) | 0.0000 | 1.1504 | 0.0190 |
| Female | 0.0000 | NA | 0.0000 |
| Insurance Status |  |  |  |
| Private Health Insurance | 0.0000 | NA | 0.0000 |
| Department of Veteran Affairs | 0.0000 | NA | 0.0000 |
| Medicare | 0.0000 | NA | 0.0000 |
| Other | 0.0000 | NA | 0.0000 |
| **Cardiac Risk Factors** |  |  |  |
| Smoking | 0.0000 | NA | 0.0000 |
| Diabetes Mellitus | 0.0000 | NA | 0.0000 |
| Hypercholesterolaemia | 0.0000 | NA | 0.0000 |
| Hypertension | 0.0000 | NA | 0.0000 |
| Congestive Heart Failure | 0.0000 | NA | 0.0000 |
| Previous Cardiac Procedure |  |  |  |
| CABG | 0.0000 | NA | 0.0000 |
| Valve | 0.0000 | NA | 0.0001 |
| **Other Comorbidities** |  |  |  |
| Cerebrovascular Disease | 0.0000 | NA | 0.0000 |
| Peripheral Vascular Disease | 0.0000 | NA | 0.0000 |
| Respiratory Disease | 0.0000 | NA | 0.0000 |
| Preoperative Dialysis | 0.0000 | NA | 0.0001 |
| Last Preop Creatinine (median [IQR]) | 0.0000 | 0.9045 | 0.0404 |
| **Cardiac Status at Time of Surgery** |  |  |  |
| Myocardial Infarction |  |  |  |
| Any MI | 0.0000 | NA | 0.0000 |
| Recent MI (< 21 days) | 0.0000 | NA | 0.0000 |
| New York Heart Association Class |  |  |  |
| I | 0.0000 | NA | 0.0000 |
| II | 0.0000 | NA | 0.0000 |
| III | 0.0000 | NA | 0.0001 |
| IV | 0.0000 | NA | 0.0000 |
| Canadian Cardiovascular Society Class |  |  |  |
| No Angina | 0.0000 | NA | 0.0000 |
| I | 0.0000 | NA | 0.0001 |
| II | 0.0000 | NA | 0.0000 |
| III | 0.0000 | NA | 0.0000 |
| IV | 0.0000 | NA | 0.0000 |
| Number of Diseased Coronary Vessels |  |  |  |
| 0 | 0.0000 | NA | 0.0000 |
| 1 | 0.0000 | NA | 0.0000 |
| 2 | 0.0000 | NA | 0.0000 |
| 3 | 0.0000 | NA | 0.0000 |
| LVEF Estimate < 30% | 0.0000 | NA | 0.0000 |
| Cardiogenic Shock | 0.0000 | NA | 0.0000 |
| Arrhythmia | 0.0000 | NA | 0.0000 |
| Infective Endocarditis | 0.0000 | NA | 0.0000 |
| **Preoperative Interventions** |  |  |  |
| Immunosuppressive Therapy (< 30 days) | 0.0000 | NA | 0.0000 |
| Inotropes (< 24 hours) | 0.0000 | NA | 0.0000 |
| Nitrates (< 24 hours) | 0.0001 | NA | 0.0001 |
| Anticoagulation (< 24 hours) | 0.0000 | NA | 0.0000 |
| Steroids (< 24 hours) | 0.0000 | NA | 0.0000 |
| Aspirin (< 7 days) | 0.0000 | NA | 0.0000 |
| Clopidogrel (< 7 days) | 0.0000 | NA | 0.0000 |
| Other antiplatelets (< 7 days) | 0.0000 | NA | 0.0000 |
| Preop Resuscitation (<1 hour) | 0.0000 | NA | 0.0000 |
| **Surgical Factors** |  |  |  |
| Urgency of Surgery |  |  |  |
| Elective | 0.0000 | NA | 0.0000 |
| Urgent | 0.0000 | NA | 0.0000 |
| Emergency | 0.0000 | NA | 0.0001 |
| Salvage | 0.0000 | NA | 0.0000 |
| Type of Surgery |  |  |  |
| Isolated CABG | 0.0001 | NA | 0.0001 |
| Isolated Valve Procedure | 0.0000 | NA | 0.0000 |
| Combined CABG/Valve | 0.0000 | NA | 0.0000 |
| Other Cardiac Procedure | 0.0000 | NA | 0.0000 |
| **Intra-/Peri-operative Processes** |  |  |  |
| Cardiopulmonary Bypass |  |  |  |
| CPB Used | 0.0000 | NA | 0.0000 |
| Cumulative CPB Time (median [IQR]) | -0.0001 | 0.9247 | 0.0228 |
| Cumulative Cross Clamp Time (median [IQR]) | 0.0000 | 0.9424 | 0.0236 |
| Intra-op Antifibrinolytic Use | 0.0000 | NA | 0.0000 |
| Intra-aortic Balloon Pump | 0.0001 | NA | 0.0001 |
| PRBC |  |  |  |
| Transfused | 0.0000 | NA | 0.0000 |
| NOVO |  |  |  |
| Transfused | 0.0000 | NA | 0.0000 |

**References**

1. VanderWeele TJ, Ding P. Sensitivity Analysis in Observational Research: Introducing the E-Value. Ann Intern Med. 2017;167(4):268-74.
