# Supplementary material for: Platelet versus fresh frozen plasma transfusion for coagulopathy in cardiac surgery patients
Source: PLoS One. 2024 Jan 17;19(1):e0296726. doi: 10.1371/journal.pone.0296726 (PMC10793891; doi:10.1371/journal.pone.0296726)
Supplement: Attachment — Submitted filename: Response to Reviewers.docx [file pone.0296726.s002.docx]

**PLOS One**

**Revisions Requested**

**Suggested Response Document**

**Editor**

**Comment 1**: Please ensure that your manuscript meets PLOS ONE's style requirements, including those for file naming.

**Response**: The manuscript has been updated to comply with style requirements.

**Comment 2**: We note that you have provided funding information that is not currently declared in your Funding Statement. However, funding information should not appear in the Acknowledgments section or other areas of your manuscript. We will only publish funding information present in the Funding Statement section of the online submission form.

Please remove any funding-related text from the manuscript and let us know how you would like to update your Funding Statement. Currently, your Funding Statement reads as follows:

“The author(s) received no specific funding for this work.”

Please include your amended statements within your cover letter; we will change the online submission form on your behalf.

**Response**: The authors received no specific funding for the completion of this work. We have adjusted the Acknowledgement section to remove all references to funding.

**Reviewer #1**

**Comment 1**: Line 29. Please replace "nay" with "may".

**Response**: This has been updated.

**Comment 2**: In the introduction section, authors should also briefly mention red blood cell transfusion as a possible further strategy in bleeding patients. In fact, in contrast to FFP and PLTS, robust evidence exist on the targets to aim for red blood cell transfusion. Please discuss and cite doi: 10.1053/j.jvca.2023.08.001.

**Response**: We agree that packed red blood cell (PRBC) transfusion is typically the main strategy in addressing the bleeding patient. While our study is specifically interested in non-PRBC transfusion, we have included the suggested reference with a brief clarification statement in the Introduction as below.

*While transfusion guidelines for packed red blood cells (PRBC) do exist and are based on robust evidence, practice guidelines concerning FFP and PLTS are generally lacking.*

**Comment 3:** Why did authors exclude the most recent data from 2022? Please explain.

**Response**: Data from 2022 patient admissions is not yet available in the latest version of the ANZSCTS database

**Comment 4**: Why were patients from 2001 to 2005 excluded?

**Response**: We have corrected an error in the timeframe stated over which the ANZSCTS database collected patient information. This has been amended to 2005-2021 throughout the manuscript. There is no patient data available prior to 2005.

**Comment 5**: Please report the secondary outcomes in the main text, leaving only the definitions in the supplementary material.

**Response**: The secondary outcomes investigated are now stated in the main text as requested.

**Comment 6**: Table 2 is not clear, as no data is provided on the 2 groups, but only the RR. For this reason, it is not possible to determine which group had the best outcomes only by looking at the Table. Please modify.

**Response**: Thank you for this comment. Table 2 has been updated to include descriptive statistics regarding cohort outcomes.

**Reviewer #2**

**Comment 1**: The choice of comparing « Platelets only » and « FFP only » patients intuitively tells us that those groups were not comparable to start with. Using a newly developed sophisticated statistical method, the authors convincingly states that the model was able to litteraly produce two groups of « comparable patients », close to mimicking a randomized trial of treament A vs B. Not being a statistician, I personnaly would challenge the authors and request a formal review by a statistical expert on the relevance of the model used herein.

**Response**: Thank you for seeking clarification of our statistical methodology. Entropy weighting is a type of Inverse Probability Weighting (IPW) that can be used to create two cohorts with balanced covariate distribution. The statistical methods outlined in our study have been conceptualised and formally reviewed prior to submission by our named author Noah Greifer, who is a data science specialist, statistical consultant and programmer for the Institute for Quantitative Social Science (IQSS) Harvard University, USA.

**Comment 2**: In addition, looking into the details, cryoprecipitate was used in 27% of the patients receiving « platelets only ». Cryoprecipitate being a frozen blood product prepared from fresh frozen plasma, it is rich in plasma proteins such factor 8, fibrinogen, factor 13, von Willebrand factor, and fibronectin.

Instead of perfoming substudies on the CABG subgroup or the most recent surgical group, it would be interesting to exclude from the « platelets only » group all patients having received cryoprecipitate and re-run the statistics.

**Response**: Given cohorts were balanced after entropy weighting, any effects of cryoprecipitate transfusion were balanced between cohorts, and thus not significant.

However, we have now also included a sensitivity analysis including only patients who were not exposed to cryoprecipitate for comparison. The results are provided in the revised manuscript and online supplement. The results are concordant with the main cohort study, and an additional comment has been included in the Results section of the manuscript to highlight this, as below.

*The primary outcomes in our three sensitivity analyses were concordant with the main cohort primary outcome. FFP was associated with increased operative mortality in the CABG-only cohort (RR, 1.71; 95% CI, 128 to 2.27; P<0.001), in the 2017 through 2021 cohort (RR, 1.59; 95% CI, 1.20 to 2.10; P<0.001) and in the cohort not exposed to cryoprecipitate (RR, 1.62; 95% CI, 1.36 to 1.94; P<0.001).*

**Comment 3**: How the authors can reconcile that the FFP group had higher chest drain output, but that the PLTS group returned more frequently to the OR for bleeding ?

**Response**: Thank you for this comment. A statement exploring this interesting topic has been added to the Discussion, shown below.

*Interestingly, FFP was associated with higher 4-hour chest drain output while PLTS were associated with higher rates of return to theatre for bleeding. FFP transfusion may contribute to a hypervolaemic state and subsequent increased 4-hour chest drain output. While chest drain output may be used clinically as a surrogate for bleeding severity, the decision to return to theatre for resternotomy, on average, occurs 8 hours post admission to ICU postoperatively.[33] As such, the association between 4 hour chest drain output and return to theatre for bleeding is not clear.*

**Comment 4**: As a detail, in all tables provided, information of the « reference group » for the RR description should be clearly stated, thouth this information can be found in the text.

**Response**: Please see response to Reviewer 1, Comment 6

**Comment 5**: Large databases often lack granularity secondary to insufficient data registration or missing data at the time of extraction : in the present study, nearly 10% of missing data on the topic of « use of intra-operative antifibrinolytic agent ».

**Response**: I believe the full comment from the reviewer has been cut off. However, we have provided justification for our strategy in relation to missing data below.

The ANZSCTS database is one of the world’s largest, most granular, prospectively collated cardiothoracic surgery databases. All missing data included in the study were assumed Missing at Random, and imputed using Multivariate Imputation by Chained Equations. Current literature suggests that Multivariate Imputation reduces bias in Missing at Random data, even with large degrees of missingness. A citation for this (https://doi.org/10.1016/j.jclinepi.2019.02.016) has been included in the references of the manuscript.

*Missing data (S1 File), defined as less than 20% missingness, were imputed using Multivariate Imputation by Chained Equations using 20 imputations and 40 iterations.[18, 19]*
